# Molecular Survey and Genetic Characterization of Hop Stunt Viroid (HSVd) in Fruit Trees in Kazakhstan

**DOI:** 10.3390/v17121547

**Published:** 2025-11-26

**Authors:** Leila T. Nadirova, Gulshan E. Stanbekova, Anna S. Nizkorodova, Ruslan V. Kryldakov, Bulat K. Iskakov, Andrey V. Zhigailov

**Affiliations:** M. Aitkhozhin Institute of Molecular Biology and Biochemistry, 86 Dosmukhamedov str., Almaty 050012, Kazakhstan; leila.nadirova@gmail.com (L.T.N.); gulshanst@yahoo.com (G.E.S.); anna_niz@yahoo.com (A.S.N.); kryldakov@yahoo.com (R.V.K.); bulat.iskakov2@gmail.com (B.K.I.)

**Keywords:** HSVd, viroid, RNA secondary structure, stone fruit tree, phylogenetic analysis, SNP

## Abstract

Hop stunt viroid (HSVd) infects a variety of natural hosts, including fruit trees, leading to significant economic losses worldwide. This survey aimed to assess the incidence of HSVd in fruit trees in southern Kazakhstan. Out of 482 fruit trees examined, 28 (5.81%) were found to be infected with HSVd. The incidence was significantly higher in stone fruit trees (8.15%; 22/270) compared to pome fruit trees (2.82%; 6/212; *p* = 0.0133). Apricots had the highest infection rate at 12.66% (10/79), while pears had the lowest rate at 2.08% (1/48). Fifteen of the identified viroids were cloned for full-genome sequencing. Sequence analysis revealed a high percentage of nucleotide sequence similarity (99–100%) among the Kazakhstani HSVd isolates, suggesting a possible unique origin for the infection. We also identified several SNPs of HSVd that have not been previously documented. A phylogenetic analysis indicated that the Kazakhstani HSVd variants clustered together in a separate group, distinct from the five known groups of HSVd. This potentially new group displayed differences in its predicted secondary structure compared to other viroid groups. These findings emphasize the need for the development of effective control measures for HSVd and other viroids affecting fruit trees.

## 1. Introduction

Viroids are small, single-stranded, covalently closed circular RNAs that do not code for proteins [1]. They infect higher plants and can cause diseases, leading to significant crop losses [2]. Currently, viroids are classified into two families based on their biochemical and structural characteristics: *Pospiviroidae* and *Avsunviroidae* [3]. Members of the *Pospiviroidae* family have rod-like or quasi-rod-like conformations and replicate using DNA-dependent RNA polymerase II within the nuclei of infected cells [4]. In contrast, members of the *Avsunviroidae* family possess a catalytic hammerhead ribozyme in their sequence, which allows for self-cleavage. They replicate through a symmetrical rolling-circle mechanism in chloroplasts [4].

Hop stunt viroid (HSVd), scientifically known as *Hostuviroid impedihumuli*, is the type species of the genus *Hostuviroid* within the family *Pospiviroidae* [5,6,7]. HSVd is found worldwide [6,7] and has a wide natural host range, including most stone fruit trees [5]. It has been identified as the causal agent of several plant diseases, including hop stunt [8], dappled fruits in plums and peaches [9], yellow spots and fruit degeneration in apricots [10,11], cachexia in citrus [12], and pale fruits in cucumbers [13]. In fruit trees, HSVd infections can lead to reductions in both fruit yield and quality [12]. However, in many instances, an infection may remain asymptomatic [5,14].

The circular RNA of the *Pospiviroidae* family consists of five functional and structural domains: the terminal left (TL), pathogenicity (P), variable (V), terminal right (TR), and central (C) domains [15]. The TL domain of HSVd contains a terminal conserved hairpin (TCH), which is essential for the replication process of *Pospiviroidae* [16]. The P domain is believed to play a role in modulating the pathogenicity and virulence of the viroid [17,18]. The C domain is crucial for viroid replication, serving as a processing site for the oligomeric forms of (+)-stranded linear replication intermediates, converting them into mature circular monomers [19,20]. The upper strand of the C domain, along with two flanking inverted repeats, can rearrange to form a hairpin I (HPI), which exists only as an intermediate in a thermodynamically metastable state during replication [15,21]. The HPI hairpin is crucial for processing linear replication intermediates into mature circles of pospiviroids [15,21]. Additionally, the C domain of *Pospiviroidae* has been reported to play a role in viroid pathogenicity [22]. The V domain shows the greatest sequence variability among closely related viroids but includes hairpins that are essential for the transcription of (–)-stranded replication intermediates in pospiviroids [15,23]. Furthermore, the TR domain is vital for the intracellular movement, cell-to-cell movement, and long-distance movement of pospiviroids [24]. Most of the polymorphisms found among HSVd isolates are concentrated in the P and V domains [15].

Kazakhstan is a landlocked country situated in the center of Eurasia, bordered by Russia, China, Uzbekistan, Kyrgyzstan, and Turkmenistan. Currently, there is no available data on the spread of HSVd in Kazakhstan [7]. Studies have reported HSVd occurrence in regions of Russia [25] and China [8,26,27], bordering Kazakhstan. Globalization in agriculture has led to increased demand for the trade of propagation materials, following the significant removal of geographic borders, and poses a substantial risk of introducing previously uncommon regional pathogens. Furthermore, highly pathogenic plant viruses and viroids, which have a limited distribution, can spread over vast areas due to increasing globalization, potentially causing significant economic damage in various countries. Information on the distribution of viroids in Central Asia is sparse, making it essential to study their prevalence and genetic diversity in economically important crops. The only report in Kazakhstan that pertains to the detection and sequencing of the HSVd genome involves grapevines (*Vitis vinifera*) and was conducted using next-generation sequencing (NGS) on the Oxford Nanopore platform [28].

This study aims to determine the incidence of HSVd in fruit trees in southern Kazakhstan and to analyze its molecular genetic characteristics. This research is part of a government program focused on studying viral and viroid diseases that affect fruit trees. To the best of our knowledge, the survey and molecular characteristics of HSVd in Kazakhstan’s fruit trees have not been explored previously. This study represents the first report on the presence of HSVd in fruit trees in Kazakhstan.

## 2. Materials and Methods

### 2.1. Study Area

Kazakhstan has a large territory, mainly occupied by deserts and semi-deserts. Agriculture in these circumstances is extremely risky. The coldest month in Kazakhstan is January, while the warmest is July. However, the foothill zone of southern Kazakhstan has favorable conditions for irrigated agriculture and the cultivation of fruit trees. About half of the groundwater resources are concentrated in southern Kazakhstan, and more than 90% of the country’s overall irrigation water is used here [29]. Fruit trees are predominantly cultivated in this region of Kazakhstan [30], particularly in the Turkistan and Almaty oblasts. These regions lead the country in fruit production, with annual yields of 360,625.7 centners and 132,748.0 centners, respectively. Together, they account for over 60% of Kazakhstan’s total fruit output [29].

**Figure 1:**
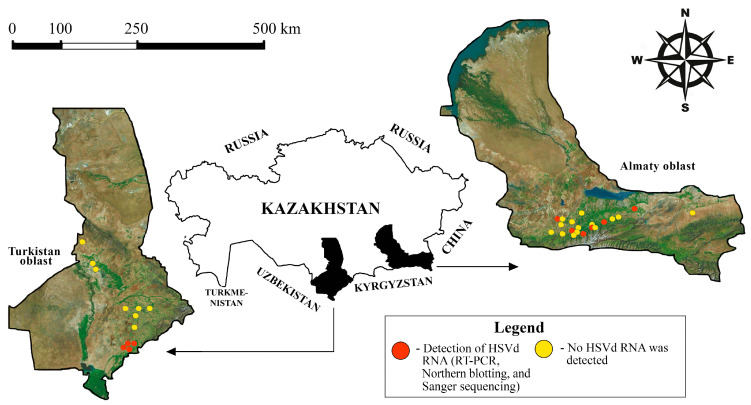
The geographic locations of the sampling sites included in this study. The map was prepared using the Earth Satellite Map online service tool [32].

Among all regions, the Turkistan oblast boasts the warmest climate. In July, average daily temperatures here range from 18 °C to 33 °C, and in January, they range from −5 °C to 3 °C. In the southern part of the oblast, severe frosts (below −20 °C) are rare [31]. The Almaty oblast experiences slightly colder temperatures. For instance, in Almaty, which is the center of the country’s horticulture, average daily temperatures in July range from 16 °C to 29 °C, while in January, they range from −10 °C to −2 °C; here, severe frosts occur regularly [31]. As part of a state program aimed at studying fruit tree viroids, a survey of HSVd was conducted in these oblasts (Figure 1).

### 2.2. Plant Material and RNA Isolation

A total of 482 fruit trees were tested for the presence of HSVd. The varieties included apple, pear, apricot, peach, cherry, sweet cherry, plum, blackthorn, and cherry plum trees. These samples were collected from 32 locations across six districts in the Almaty oblast and six districts in the Turkistan oblast of Kazakhstan (Supplementary Table S1). Due to climatic conditions, citrus fruits cannot be grown outdoors in Kazakhstan; therefore, these trees were not included in the survey. The sample collection periods were from July 2023 to September 2023 and from June 2024 to September 2024.

We used a semi-targeted approach for sampling. Seven locations, including the “Botany Garden,” “Dendarium,” “Tastybulak,” “Atbulak,” “Sayram,” “Jemisty,” and “Chundzha,” were selected for their excellent representation of fruit trees. These areas served as nurseries, arboretums, gardens, or orchards. The remaining 25 localities were randomly selected from 816 settlements within the territory of fruit tree cultivation in two selected oblasts of the country using the RANDBETWEEN function in MS Excel 2016, which generates random numbers with a normal distribution. We used this function to minimize the subjective selection of site locations. In one locality, a maximum of three different sampling sites were used. At certain collection sites, samples were collected regardless of the symptoms displayed. At one collection site, we selected no more than five stone fruit trees of the same species.

From each tree, we collected three to five leaves from various parts of the crown. The geographic locations of the selected sampling sites are shown in Figure 1. We extracted total RNA from fresh or frozen leaf samples (0.3 g) using the cetyltrimethylammonium bromide (CTAB) method [33]. The extracted RNA was stored at −80 °C until further analysis.

### 2.3. Reverse Transcription, PCR, and cDNA Cloning

To detect HSVd RNA, we used a two-step RT-PCR method. Primers hsvdf8 (5′-CGGATCCTCTCTTGAGCC-3′) and hsvdr8 (5′-CGGCAGAGGCGCAGATAGAACA-3′), which amplify the full genome (294–303 nucleotides), were used as specific primers. HSVd-F2 (5′-GGGGCAACTCTTCTCGGAATCC) and HSVd-R2 (5′- GGGGCTCCTTTCTCAGGTAAGTA) were used as additional specific primers. All these primers were designed based on reference HSVd GenBank sequences (GenBank: AB742224, AF131248, AF213489, AF462156, AJ297831, D13765, DQ362903, EF151290, EF523827, EU365348, FJ984562, GQ995465, KC771550, KC771556, KJ466327, KY445746, MF774874, MT769771, MW015998, MZ196517, ON669246, OP885280, OR924343, OR892431, PQ059409, PQ736858, X06719, X07405, X87928, Y08438, and Y09347). The reference sequences were chosen after conducting a preliminary phylogenetic analysis of all complete HSVd genomes available in GenBank. This analysis utilized the online Basic Local Alignment Search Tool (BLAST) program (release 264.0) [34]. From each clade of the resulting HSVd phylogenetic tree, a representative sequence was selected based on the availability of information in GenBank regarding the source, host, collection date, and county. Preference was given to isolates collected more recently.

Primers hsvdf8 and hsvdr8, which amplified full HSVd genomes, were used to estimate the incidence, clone, and sequence the complete genomes of HSVd. If multiple positive HSVd samples were found at a single sampling site for the same fruit species, as a rule, only one was chosen for cloning and sequencing. Selected amplicons underwent direct Sanger sequencing to verify their identity as HSVd. Following this confirmation, the amplicons were cloned, enabling the sequencing of the complete HSVd genomes. To determine the nucleotide sequence of the viroids in the regions targeted by the primers hsvdf8 and hsvdr8, RNA samples that tested positive for HSVd RNA were further analyzed using the primers HSVd-F2 and HSVd-R2. These primers also amplified the full HSVd genomes. In this instance, the amplification products were sequenced directly, eliminating the need for cloning.

At the first step, the cDNA was synthesized from the RNA using Maxima Reverse Transcriptase (Thermo Fisher Scientific, Waltham, MA, USA) with random hexamer primers (Thermo Fisher Scientific, Waltham, MA, USA), according to the manufacturer’s instructions.

Amplification was performed using Pfu DNA polymerase (Thermo Fisher Scientific, Waltham, MA, USA) following the manufacturer’s instructions. Each 25 µL reaction included 2.5 µL of cDNA, 2.5 µL of 10× Standard Pfu Buffer with MgSO_4_ (Thermo Fisher Scientific, Waltham, MA, USA), 0.2 mM dNTPs (Thermo Fisher Scientific, Waltham, MA, USA), 0.24 µM of each primer, 1.25 U of Pfu DNA polymerase (Thermo Fisher Scientific, Waltham, MA, USA), and nuclease-free water to reach the final volume. The thermal cycling conditions comprised an initial denaturation step at 95 °C for 2 min, followed by 35 cycles of denaturation at 95 °C for 30 s, annealing at 57 °C for 30 s, and extension at 72 °C for 1 min. This was concluded with a final incubation at 72 °C for 5 min. The PCR products were analyzed using 1.8% TBE agarose gel electrophoresis and visualized under UV light.

The selected PCR products with expected sizes were purified from the gel using a QIAquick Gel Extraction Kit (Qiagen, Germantown, MD, USA). Subsequently, TA cloning into the pBluescript SK II(+) vector was performed [35]. At least two DNA clones from each isolate were Sanger sequenced in both directions using standard M13 forward and reverse primers.

### 2.4. Northern Blotting

The HSVd cDNA, cloned in the pBluescript II SK(+) vector, was used to synthesize a positive-strand-specific probe using T7 RNA polymerase. The pBS-SK/PP857835 construct, which contains the full-length HSVd sequence (GenBank: PP857835), was linearized with the *Sac*I restriction enzyme (Thermo Fisher Scientific, Waltham, MA, USA). Following this, the construct was transcribed using the DIG RNA Labeling Kit (SP6/T7) (Roche Diagnostics GmbH, Mannheim, Germany) according to the manufacturer’s instructions. RNA samples (300–500 ng) were denatured in a solution of formamide and formaldehyde by heating at 65 °C for 10 min. They were then chilled on ice and loaded onto a denaturing gel composed of 1% agarose and 1.25% formaldehyde. Electrophoresis was performed at a constant voltage of 150 V in 1× MOPS buffer, at a rate of 8 V/cm for 10 min, followed by 6 V/cm for 1.5 to 2 h. Before transferring the RNA to a nylon membrane, the gel was briefly rinsed with RNase-free water and soaked in 10× SSC (1.5 M NaCl, 0.15 M trisodium citrate, pH 7.0) for 20 to 30 min. The gel was then placed in a capillary transfer apparatus, and RNA was transferred to a Hybond N+ nylon membrane (GE Healthcare Amersham, Little Chalfont, UK) overnight by capillary action using 10× SSC.

After transfer, the membrane was rinsed with 70% ethanol and crosslinked with 1200 J of 254 nm shortwave UV light. Next, prehybridization of the membrane was carried out at 68 °C for 1 h in DIG-Easy Hyb solution (Roche Diagnostics GmbH, Mannheim, Germany). This was followed by hybridization with a DIG-labeled RNA-probe (50 ng/mL) in the same solution at 68 °C overnight. The blot was washed twice in 2× SSC with 0.1% SDS at 68 °C for 20 min and then twice in 0.1× SSC with 0.1% SDS at 68 °C for an additional 20 min. After washing, the blot was incubated for 1 h in 2% blocking buffer, followed by another 1 h incubation with Anti-Digoxigenin-AP Fab fragments (Roche Diagnostics GmbH, Mannheim, Germany) at a dilution of 1:5000 in blocking buffer. The membrane was then washed twice for 10 min in washing buffer and equilibrated for 5 min in detection buffer before applying the chemiluminescent substrate CDP-Star AP Substrate (Novagen Specialty Ltd., London, UK) according to the manufacturer’s instructions. Finally, the chemiluminescent signals were captured on X-ray film (Agfa HealthCare NV, Mortsel, Belgium).

### 2.5. Sequencing and Phylogenetic Analysis

The cloned HSVd cDNAs were sequenced in both directions using the BigDye Terminator v3.1 Cycle Sequencing Kit (Applied Biosystems, Carlsbad, CA, USA), according to the manufacturer’s recommendations, and analyzed using a 24-capillary ABI 3500XL Genetic Analyzer (Applied Biosystems, Foster City, CA, USA). If multiple positive HSVd samples were collected from a single site and host, only one sample was selected for cloning and sequencing.

The BLAST program (version 264.0) was utilized to compare the resulting nucleotide sequences with those available in the NCBI GenBank database [34] and to assess the statistical significance of the matches. Multiple sequence alignment was conducted using the MUSCLE algorithm. Phylogenetic relationships among the analyzed isolates were determined using maximum likelihood algorithms and a model selected based on the lowest Bayesian Information Criterion score. The Molecular Evolutionary Genetics Analysis (MEGA) X software, version 10.1.8 (The Pennsylvania State University, State College, PA, USA) [36], was employed for phylogenetic analysis. The bootstrap method, with 1000 replicates, was used to evaluate the reliability of the tree topologies [37].

The sequences reported in this study are available in the GenBank database [34] under the accession numbers PP857835, PV400859–PV400864, PV053182–PV053186, and PX516284–PX516286.

### 2.6. Prediction of Secondary Structures of Viroid RNAs

The minimum free energy of secondary structures of the HSVd variants was predicted using the online web tool RNAstructure version 6.0.1 [38,39].

## 3. Results

### 3.1. Samples

Overall, leaf samples from 482 fruit trees from 44 sites in 32 locales across the two oblasts of southern Kazakhstan were at our disposal (Figure 1 and Supplementary Table S1). Most trees showed no conspicuous symptoms. However, two trees exhibited delayed foliation; one of them did not produce leaves the following growing season and died. Additionally, one peach tree exhibited cracked bark and fruit deformation.

### 3.2. Identification of HSVd from Fruit Trees

**Figure 2:**
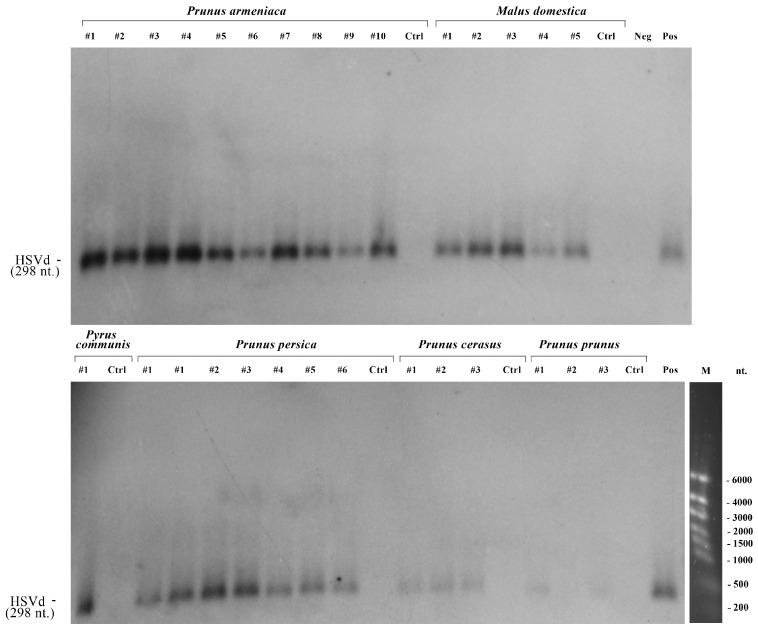
Northern-blot hybridization testing of selected RNA samples. Neg—negative control (water); Ctrl—control samples (RT-PCR negative tree); Pos—positive control, RT-PCR positive sample confirmed by sequencing method (HSVd-KZ-AN isolate); M—RiboRuler High Range RNA ladder (Thermo Fisher Scientific, Waltham, MA, USA), 1% agarose/1.25% formaldehyde gel line.

Using HSVd-specific primers, we examined all RNA samples using conventional RT-PCR. Out of 482 trees studied, 29 tested positive for HSVd. To further confirm the infection of fruit trees with HSVd, all 29 RT-PCR positive samples were tested using the Northern blotting method. The results are presented in Figure 2. Out of the 29 RT-PCR positive samples, 28 confirmed the presence of HSVd RNA. There were no positive results from the Northern-blot tests corresponding to negative results from the RT-PCR (Figure 2). So, one RT-PCR positive sample from a plum was not confirmed by Northern blotting. Therefore, the overall prevalence of HSVd in fruit trees in Kazakhstan is 5.8% (28/482; 95% CI = 3.9–8.3%). The highest infection rate was in apricot trees (12.7%; 10/79), followed by peach (9.2%; 7/76), cherry (4.6%; 3/65), plum (4.0%; 2/50), apple (3.0%; 5/164), and pear (2.1%; 1/48) trees. The incidence of HSVd was significantly higher in stone fruit trees (8.15%; 22/270) compared with that in pome fruit trees (2.82%; 6/212; χ^2^ = 6.13; *p* = 0.0133).

The summarized data for RT-PCR, Northern blotting, and HSVd sequencing are presented in Table 1.

Out of the 28 trees infected with HSVd, only 3 (10.7%, 95% CI = 2.3–28.2%) exhibited at least one of the following symptoms: bark cracking, death within a year, delayed foliation, or fruit deformation (Table 2). The remaining 25 HSVd-positive trees showed no symptoms. The most notable symptoms were seen in peach trees from the Almaty oblast. One possible explanation for this observation is that the climate in this oblast of Kazakhstan is less favorable for peaches, as it endures harsher winters compared to the Turkistan oblast [31]. Additionally, co-infection with other peach pathogens may also play a role in the severity of these symptoms. Unfortunately, we did not obtain the complete virome from samples of the three symptomatic trees. Furthermore, we did not conduct experiments involving the mechanical inoculation of control trees with Kazakhstani HSVd isolates. As a result, we cannot eliminate the possibility of co-infection with other pathogens, which means the observed clinical manifestations might not be solely due to HSVd.

### 3.3. Phylogenetic Analysis of the Identified HSVd Variants

**Figure 3:**
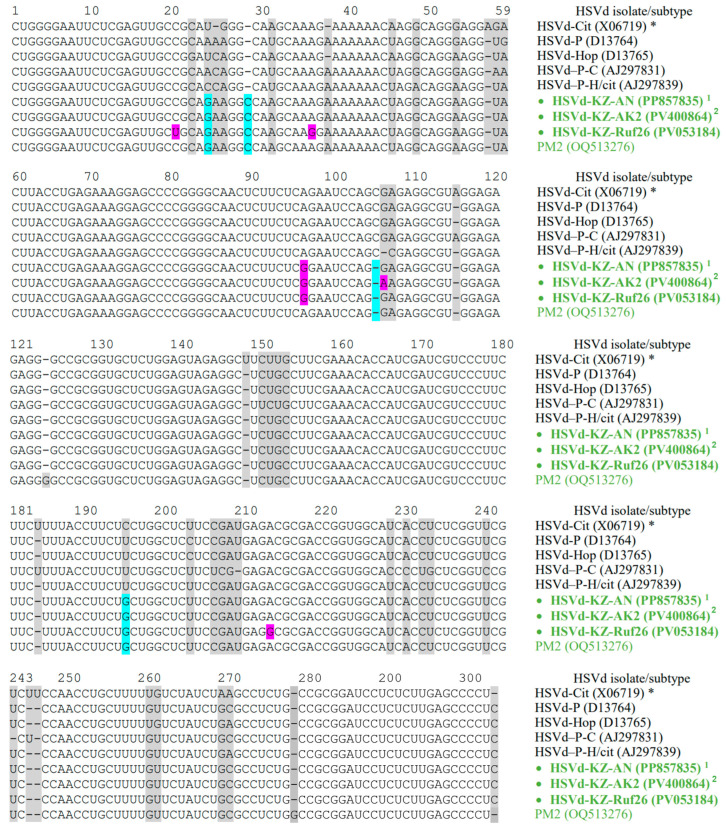
The alignment of cloned whole-genome sequences of HSVd from southeastern Kazakhstan. The sequences of the representative strains of HSVd subtypes are shown for comparative purposes. Nucleotide variations are indicated with gray boxes. Nucleotide variations identified only in Kazakhstani isolates are indicated with pink boxes. Nucleotide variants characteristic of newly suggested group are indicated with blue boxes. The names of HSVd isolates included to the newly suggested group are highlighted in green. Kazakhstani HSVd isolates determined in this study are marked with a circle (●). The nucleotide numbering corresponds to the reference HSVd genome marked with an asterisk (*) (GenBank: X06719) [40]. ^1^ The same nucleotide sequences are in found isolates HSVd-KZ-KEp1 (GenBank: PV053182), HSVd-KZ-KEp3 (GenBank: PV053183), HSVd-KZ-SAY (GenBank: PV400861), HSVd-KZ-ShM (GenBank: PV400859), HSVd-KZ-BES (GenBank: PV400860), HSVd-KZ-Sar72 (GenBank: PV053185), HSVd-KZ-Jem35 (GenBank: PV053186), HSVd-KZ-SAR (GenBank: PV400862), HSVd-KZ-AK1 (GenBank: PV400863), HSVd-KZ-Jem2 (GenBank: PX516286), and HSVd-KZ-AK3 (GenBank: PX516284). ^2^ The same nucleotide sequences are in found isolates HSVd-KZ-AK4 (GenBank: PX516285).

To achieve complete coverage of the HSVd genomes, we cloned fifteen of the generated amplicons into the pBluescript SK II(+) vector using the TA-cloning method. An analysis of the complete nucleotide sequences of the cloned HSVd variants, conducted using the dideoxy sequencing technique, revealed that all the cloned amplicons were comparable with those in the HSVd genome. The homology among all Kazakhstani HSVd variants was notably high, ranging from 99.0% to 100%. We identified three new variants; twelve HSVd isolates had identical full genome nucleotide sequences (Table 2). We identified several single-nucleotide polymorphisms (SNPs) of HSVd that had not been documented previously (Table 2). One of them, namely the A96G mutation, was found in all Kazakhstani HSVd isolates (Table 2). The multiple sequence alignment of the full-length HSVd clones is presented in Figure 3.

**Figure 4:**
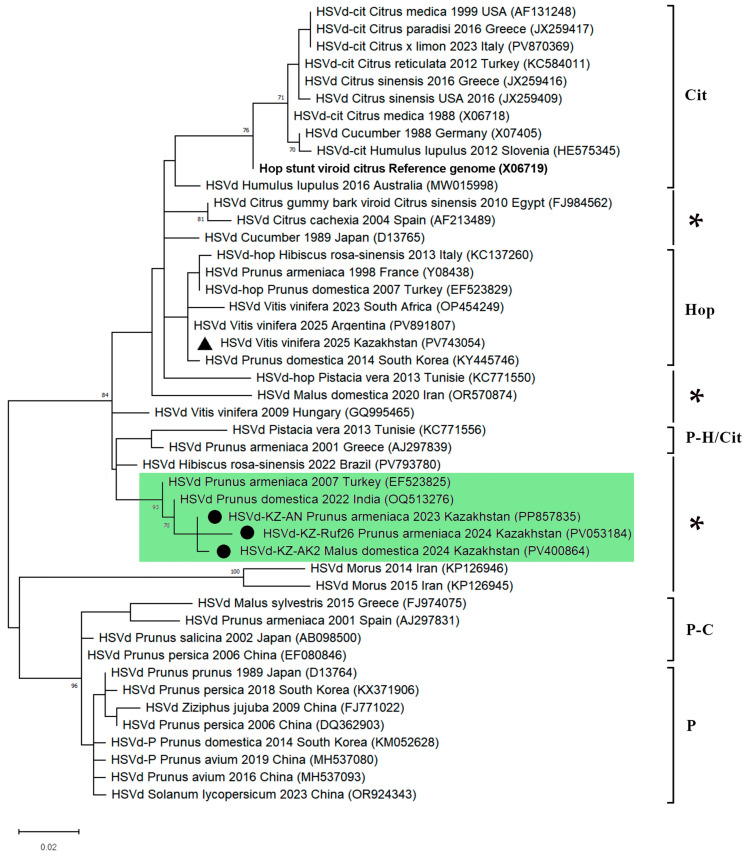
Phylogenetic analysis based on the full-genome sequences of HSVd isolates. A maximum-likelihood phylogenetic tree was constructed in MEGA X from the alignments of 15 complete HSVd sequences generated in this study and 44 database sequences, using Kimura’s two-parameter method (K2+G model) [47]. The tree is drawn to scale, with the branch lengths representing the number of substitutions per site. The percentage of trees in which the associated taxa were clustered is shown next to the branches. Bootstrap values that have high confidence (70 or higher) are displayed exclusively. The GenBank accession numbers are shown in parentheses. The Kazakhstani HSVd isolates determined in this study are marked with a black circle. The Kazakhstani HSVd isolate, previously identified in grapevine using the NGS method (GenBank: PV743054) [28], is marked with a black triangle. The reference genome (GenBank: X06719) is indicated in bold [40]. The five phylogenetic groups described by Amari et al. [43] are indicated by brackets. The branches of the HSVd variants that could not be included in the known five groups are marked with an asterisks (*). Isolates of HSVd included in a potentially new group are marked with a green rectangle. Abbreviations: Cit: citrus–type; P: plum–type; Hop: hop–type; P-C: plum–citrus–type; P-H/Cit: plum–hop–citrus–type.

The representatives of three Kazakhstani HSVd variants characterized in this study and 31 representative HSVd sequences, selected based on the first phylogenetic analysis, were subjected to a second phylogenetic analysis. Using the phylogenetic grouping classification of previous reports [9,41,42,43], the HSVd variants were divided into five groups: citrus-type, plum-type, hop-type, plum-citrus-type, and plum-hop-citrus-type (Figure 4). However, two unpublished sequences from Turkey (GenBank: EF523825) and India (GenBank: OQ513276), along with Kazakhstani HSVd variants, were clustered separately, with a low mean nucleotide intragroup divergence reaching 1.84%, and could not be assigned to any of the five related HSVd phylogenetic groups (Figure 4). We believe that a sixth phylogenetic group of HSVd could therefore be reasonably recognized. Several previous studies have reported that phylogenetic analysis of HSVd sequences available in nucleotide databases [44,45], or analysis of newly sequenced sequences [46], revealed variants that could not be included in the known five groups. This suggests a potential need to revise the classification of HSVd genetic variants, especially considering the new data that has emerged over the past two decades. Given that the viroid genome is very small, consisting solely of protein-noncoding sequences and exhibiting complex structures, isolating a new group of HSVd based solely on phylogenetic analysis is quite challenging. Therefore, further research on this topic is necessary.

### 3.4. RNA Secondary Structure

**Figure 5:**
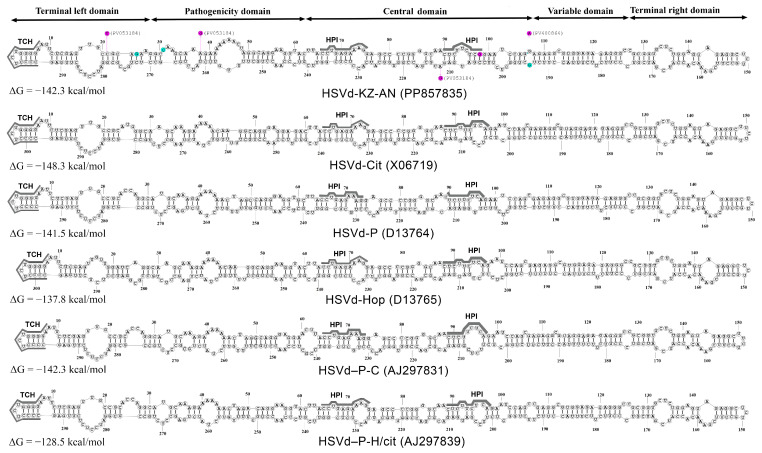
Secondary structures of hop stunt viroid. The nucleotide sequences of the Kazakhstani variants of HSVd, along with the type isolates from five major HSVd groups, were analyzed and are depicted in their secondary structures. The structures were generated using the RNAstructure version 6.0.1 prediction tool [39], employing experimental data at 26 °C as constraints. The structures with the lowest Gibbs free energy are displayed. The Kazakhstani type isolate HSVd-KZ-AN (GenBank: PP857835) is shown at the top of the figure. Specific sequence variations in two different Kazakhstani HSVd variants are plotted onto the predicted secondary structure. Nucleotide variations unique to the Kazakhstani isolates are highlighted with pink circles, while nucleotide variants characteristic of the newly proposed HSVd group are indicated with blue circles. The GenBank accession numbers for these sequences are included in parentheses. The boundaries of various HSVd domains are marked according to data from Wüsthoff and Steger [15]. Abbreviations: ΔG: the Gibbs free energy; Cit: citrus–type; P: plum–type; Hop: hop–type; P-C: plum–citrus–type; P-H/Cit: plum–hop–citrus-type; TCH: terminal conserved hairpin; HPI: the two sequence regions form hairpin I in the metastable structures of *Pospiviroidae* sequences [15].

RNA secondary structures play a crucial role in the function and evolution of viroids. Nucleotide substitutions can be preserved only if significant secondary and tertiary structures are not disrupted [2]. We identified four specific mutations characterizing the suggested new HSVd group, namely, U25G, 28_29insC, 105delC, and C195G (indicated by the blue boxes in Figure 3). Then, we analyzed the relationship between these mutations and the predicted RNA secondary structures. One representative genome from the Kazakhstani HSVd isolates (GenBank: PP857835) was subjected to the secondary structure analysis using the RNAstructure online server of Mathews Lab Home [38]. An analysis of the predicted secondary structures revealed that these mutations are located at the boundaries between the TL and P domains and the C and V domains, distinguishing them from other HSVd groups (Figure 5). The secondary structures of typical reference sequences from the five main HSVd groups are depicted in the figure for comparative purposes.

The structure of the pathogenic and central domains of the Kazakhstani HSVd isolates is distinct from those of the five main groups of this viroid (Figure 5). The A96G mutation, present in all HSVd isolates from Kazakhstan, occurs in the region that forms the HPI hairpin during the process where linear replication intermediates are converted into mature circular forms. This mutation plays a crucial role in the replication of pospiviroids (Figure 5) [15,21]. It is important to note that the SNPs characteristic of three Kazakhstani isolates, which have a different nucleotide sequence from the HSVd-KZ-AN isolate, do not lead to any changes in the predicted secondary structure of the pospiviroid. The A213G substitution in the HSVd-KZ-Ruf26 isolate (GenBank: PV053184) and the G106A substitution in the HSVd-KZ-AK2 and HSVd-KZ-AK4 isolates (GenBank: PV400864 and PX516285) occur in unpaired regions (Figure 5). Additionally, the C21U and A36G substitutions in the HSVd-KZ-Ruf26 isolate (GenBank: PV053184) are compensatory mutations that maintain the base pair at both nucleotide positions (Figure 5).

## 4. Discussion

This study found that HSVd was present in fruit trees of at least six species in southern Kazakhstan, including apricot, peach, cherry, plum, apple, and pear. The incidence of HSVd in these fruit trees was comparable to levels observed in other Asian countries [27,48,49,50,51,52,53]. We detected HSVd RNA not only in stone fruit crops but also in pome fruit trees like apples and pears, which are not typically considered primary hosts of HSVd. However, several studies have confirmed the presence of HSVd in both apple and pear trees [51,53,54,55], indicating that these fruit crops can potentially be infected by this viroid. It is important to note that the infection rate of HSVd in apple and pear trees is significantly lower than that observed in stone fruit trees. Several other studies have also reported a statistically significant difference between the HSVd infection rates in stone and pome fruit trees [51,54].

We observed variations in the distribution of HSVd in southern Kazakhstan. In the Almaty oblast, HSVd RNA was detected in fruit tree samples from four different districts, indicating a broad distribution of the viroid in this oblast. In contrast, in the Turkistan oblast, HSVd RNA was found only in the Saryagash district, located in the southern part of the oblast (Figure 1).

The sequence analysis revealed that the Kazakhstani HSVd isolates share a very high percentage of complete genome sequence similarity, ranging from 99% to 100%. This suggests a potential unique origin for the infection. In Kazakhstan, the planting material for fruit trees is primarily imported from European countries and Russia [29]. However, the nucleotide sequences of the Kazakhstani HSVd isolates showed the highest similarity to those found in Turkey and India (Figure 4). It is also important to note that a genetic variant of HSVd, previously identified in grapes from Kazakhstan [28], has been classified into the Hop group based on phylogenetic analysis (Figure 4). This indicates that multiple strains of HSVd are present and circulating within Kazakhstan.

The phylogenetic analysis and the predicted secondary structure of viroids indicate that the Kazakhstani HSVd isolates cannot be classified into any of the five currently recognized HSVd groups. Based on the results of these analyses, we propose establishing a new, distinct group of HSVd to encompass the identified Kazakhstani isolates.

The most effective method for controlling viroid diseases is to detect viroids in plants and to exclude or eradicate infected materials [2]. In Kazakhstan, hop production is currently underdeveloped, and HSVd (Hop Stunt Viroid) is not classified as a quarantinable infectious agent within the Eurasian Economic Union (EAEU) [56]. At present, Kazakhstan lacks a unified system for monitoring and eradicating viroid infections, and regular surveillance for HSVd in plants is not conducted. In southern Kazakhstan, grapes and fruit trees that are susceptible to HSVd are widely cultivated. Our findings underscore the urgent need for regular monitoring surveys for HSVd in this region. It is critical that a national strategy for the control and eradication of HSVd infections is developed as soon as possible. To address this issue, we distributed information on the detection of HSVd to farm and nursery owners, and samples were collected for analysis. We hope that our results, along with ongoing efforts to enhance the effectiveness of HSVd infection control, will help reduce the transmission of HSVd into non-endemic areas. Additionally, the genetic characterization of HSVd isolates found in Kazakhstan may improve diagnostic methods for identifying this viroid.

This study has several limitations. It focused exclusively on fruit tree species, while HSVd can infect a wide range of hosts. To fully understand the diversity of this viroid in Kazakhstan, a more comprehensive survey is needed, including other important hosts such as hops, grapevines, cucumbers, and melons. Although the semi-targeted approach used for sampling increased the likelihood of detecting HSVd, it also resulted in a significant overestimation of the pathogen’s prevalence in the studied area. Additionally, mechanical inoculation of the indicator plant species was not conducted with the HSVd isolates.

## 5. Conclusions

In this study, we conducted a field survey to assess the incidence of HSVd in fruit trees across two oblasts in southern Kazakhstan, known for being leading producers of fruit trees in the country. During the survey, we detected, isolated, and genetically characterized HSVd for the first time in Kazakhstan. We identified several SNPs of HSVd that had not been previously reported. The findings from this research may enhance the effectiveness of diagnostic methods for detecting HSVd.

## Figures and Tables

**Table 1:** Summary data on the results of RT-PCR, Northern blot analysis, and Sanger sequencing.

No.	Locality	Species	RT-PCR Positive	Northern Blot Tested/Confirmed	Cloned and Sequenced
1	Ak-Kaiyn	peach	2	2/2	1
2	Esik	apricot	2	2/2	1
3	Saymasay	pear	1	1/1	1
4	Shamalgan	plum	3	3/2	1
5	Algabas	peach	1	1/1	1
6	Besagash	cherry	2	2/2	1
7		apricot	3	3/3	1
8	Akniet	cherry	1	1/1	1
9	Akzhar-1	apple	1	1/1	1
10		apricot	1	1/1	1
11	Akzhar-2	apple	4	4/4	1
12		peach	1	1/1	1
13	Jemisty	peach	3	3/3	1
14		apricot	3	3/3	1
15	Yntymak	apricot	1	1/1	1
Total			29	29/28	15

**Table 2:** List of the Kazakhstani HSVd isolates with some characteristics.

Isolate	Accession Number	Host	Locality	New SNPs	Symptoms ^1^
HSVd-KZ-AN	PP857835	apricot	Besagash	A96G	DF
HSVd-KZ-KEp1	PV053182	peach	Algabas	A96G	CB, FD
HSVd-KZ-KEp3	PV053183	peach	Ak-Kaiyn	A96G	DF; D
HSVd-KZ-Ruf26	PV053184	apricot	Esik	C21U, A36G, A96G, A213G	
HSVd-KZ-SAY	PV400861	pear	Saymasay	A96G	-
HSVd-KZ-ShML	PV400859	plum	Shamalgan	A96G	-
HSVd-KZ-BES	PV400860	sweet cherry	Besagash	A96G	-
HSVd-KZ-Sar72	PV053185	apricot	Yntymak	A96G	-
HSVd-KZ-Jem35	PV053186	apricot	Jemisty	A96G	-
HSVd-KZ-Jem2	PX516286	peach	Jemisty	A96G	
HSVd-KZ-SAR	PV400862	cherry	Akniet	A96G	-
HSVd-KZ-AK1	PV400863	apple	Akzhar-1	A96G	-
HSVd-KZ-AK3	PX516284	apricot	Akzhar-1	A96G	
HSVd-KZ-AK2	PV400864	apple	Akzhar-2	A96G, G106A	-
HSVd-KZ-AK4	PX516285	peach	Akzhar-2	A96G, G106A	

^1^ CB = cracks in the tree bark; D = death in a year; DF = delay in foliation; FD = fruit deformation.

## Data Availability

The nucleotide sequences reported in this paper are deposited into the NCBI GenBank database [34] under the accession numbers listed in the text.

## Supplementary Materials

The following supporting information can be downloaded at: https://www.mdpi.com/article/10.3390/v17121547/s1. Table S1: Number of fruit tree crop samples from different localities used in the HSVd survey.

## Abbreviations

The following abbreviations are used in this manuscript:

BLAST	Basic GenBank Local Alignment Search Tool
95% CI	95% confidence interval
EAEU	The Eurasian Economic Union
HSVd	Hop stunt viroid
MEGA	Molecular Evolutionary Genetics Analysis
SNP	Single nucleotide polymorphism
ΔG	The Gibbs free energy
