# Supplementary material for: Molecular Survey and Genetic Characterization of Hop Stunt Viroid (HSVd) in Fruit Trees in Kazakhstan
Source: Viruses. 2025 Nov 26;17(12):1547. doi: 10.3390/v17121547 (PMC12737526; doi:10.3390/v17121547)
Supplement: Supplementary file 1 [file viruses-17-01547-s001.zip › viruses-3970502-supplementary.pdf]

Supplementary Table S1. Number of fruit tree crop samples from different localities used in the HSVd survey

No.	Locality	Coordinates	Source					
			apple [1]	pear	apricot [2]	peach	cherry [3]	plum [4]
1	Karagaily	43.161956, 76.847858			3	5	3	
2	Tastybulak	43.172222, 76.815838	5		2	4		
3	Ak-Kaiyn	43.173749, 76.967011	5	1	5	2		
4	Botany garden	43.223860, 76.914160	5			5		
5	Ashybulak	43.487894, 77.775393	5	2				
6	Esik	43.357364, 77.458232	5		5		3	1
7	Saymasay	43.446146, 77.328007	5	2		2		
8	Karaturyk	43.556985, 77.994449	10	3	5	4	7	3
9	Boralday	43.370097, 76.872323	2	2		2		
10	Almalybak	43.219653, 76.680165	3	2	4	2	7	2
11	Zhalpaksay	43.237491, 76.682113	2	1	2	1	2	1
12	Korgauldy	43.168614, 76.760671	5			3		
13	Zhandosovo	43.164162, 76.555497	5	1	5	5	5	5
14	Shamalgan	43.366580, 76.637612	5	2	5		5	3
15	Turar	43.316341, 76.579084	30					
16	Algabas	43.271103, 76.796686				3		
17	Baibulak	43.282523, 77.191661	2	1		2		
18	Besagash	43.289931, 77.060820	5	2	5	1	5	5
19	Ile-Alatau	43.160126, 77.044712	5	5	5		3	2
20	Chundzha	43.524600, 79.474040	10	3		5		
21	Atbulak	42.345041, 69.492893	5			5	5	5
22	Rabat	42.059154, 69.516292	5	5	5	5		5
23	Badam	42.393802, 69.251972				3		
24	Akniet	41.452918, 69.142156	5	5			2	5
25	Akzhar 1/2	41.591276, 69.350059	10	5	6	2	5	
26	Jemisty	41.491492, 69.316311	10		7	5	8	8
27	Yntymak	41.393872, 69.106922	5	1	1			
28	Maydantal	43.683738, 68.012794	5		5			3
29	Sayram	42.297949, 69.716074			2	2		
30	Dendarium	42.367953, 69.612962	5	5	5	5	2	2
31	Birlik	43.333654, 68.247169				3		
32	Turkistan	43.289631, 68.345815			2		3	
	Total:		164	48	79	76	65	50

[1] Including wild Sivers' apple trees. [2] Including wild apricot. [3] Including sweet cherry. [4] Including blackthorn, cherry plum, and cultivated plum.
